# Supplementary material for: Machine Learning Accelerates Crystallization for Structure Determination
Source: Angew Chem Int Ed Engl. 2026 May 4;65(25):e1218503. doi: 10.1002/anie.1218503 (PMC13266958; doi:10.1002/anie.1218503)

checkCIF/PLATON report

Structure factors have been supplied for datablock(s) lcz022

THIS REPORT IS FOR GUIDANCE ONLY. IF USED AS PART OF A REVIEW PROCEDURE FOR PUBLICATION, IT SHOULD NOT REPLACE THE EXPERTISE OF AN EXPERIENCED CRYSTALLOGRAPHIC REFEREE.

No syntax errors found. CIF dictionary Interpreting this report

Datablock: lcz022

Bond precision:	C-C = 0.0142 A		Wavelength=1.54184
Cell:	a=11.6764(3)	b=12.0088(2)	c=12.0565(3)
	alpha=115.741(2)	beta=103.986(2)	gamma=101.171(2)
Temperature:	100 K		

	Calculated	Reported
Volume	1388.69(7)	1388.69(6)
Space group	P -1	P -1
Hall group	-P 1	-P 1
Moiety formula	C20 H8 Ag3 F18 N6 O	C15 H3 Ag3 F18 N6, C5 H5 O
Sum formula	C20 H8 Ag3 F18 N6 O	C20 H8 Ag3 F18 N6 O
Mr	1013.93	1013.93
Dx,g cm-3	2.425	2.425
Z	2	2
Mu (mm-1)	18.249	18.249
F000	962.0	962.0
F000’	966.88	
h,k,lmax	14,15,15	14,15,15
Nref	5979	5640
Tmin,Tmax	0.168,0.161	0.159,1.000
Tmin’	0.003	

Correction method= # Reported T Limits: Tmin=0.159 Tmax=1.000
AbsCorr = MULTI-SCAN

Data completeness= 0.943 Theta(max)= 78.420

R(reflections)= 0.0575(5056) wR2(reflections)= 0.1611(5640)

S = 1.062 Npar= 434

The following ALERTS were generated. Each ALERT has the format
test-name_ALERT_alert-type_alert-level.
Click on the hyperlinks for more details of the test.

Alert level C

```
PLAT042_ALERT_1_C Calc. and Reported MoietyFormula Strings  Differ     Please Check
             Calc: C20 H8 Ag3 F18 N6 O
             Rep.: C15 H3 Ag3 F18 N6, C5 H5 O
PLAT342_ALERT_3_C Low Bond Precision on  C-C Bonds ..............    0.01418 Ang.
PLAT906_ALERT_3_C Large K Value in the Analysis of Variance ......      4.953 Check
PLAT906_ALERT_3_C Large K Value in the Analysis of Variance ......      2.323 Check
PLAT911_ALERT_3_C Missing FCF Refl Between Thmin & STh/L=    0.600         36 Report
              -1  1  0,   0  1  0,   3-13  1,   2  9  1,   2-13  2,  -2-12  2,
               4  8  2,  -1-13  3,  -3-12  3,  -2-12  3,  -3-11  3,  -2 -1  3,
              -2-13  4,  -4-12  4,  -3-12  4,  -1-11  4,   0-11  4,   4-13  5,
              -4-12  5,  -3  3  5,   0  3  5,   0-14  6,  -1-13  6,  -2-12  6,
               5-12  6,   6-12  6,   6-11  6,  -8 -8  6,  -4 -4  6,  -7  3  6,
                 (    6 More Missing: see the .ckf listing file)
PLAT971_ALERT_2_C Check Calcd Resid. Dens.  1.02Ang From Ag01          1.93 eA-3
PLAT971_ALERT_2_C Check Calcd Resid. Dens.  1.08Ang From Ag03          1.83 eA-3
PLAT971_ALERT_2_C Check Calcd Resid. Dens.  1.03Ang From Ag03          1.78 eA-3
PLAT971_ALERT_2_C Check Calcd Resid. Dens.  1.07Ang From Ag02          1.72 eA-3
PLAT971_ALERT_2_C Check Calcd Resid. Dens.  1.01Ang From Ag02          1.61 eA-3
PLAT971_ALERT_2_C Check Calcd Resid. Dens.  1.19Ang From Ag01          1.61 eA-3
PLAT971_ALERT_2_C Check Calcd Resid. Dens.  1.12Ang From Ag03          1.55 eA-3
PLAT971_ALERT_2_C Check Calcd Resid. Dens.  1.36Ang From Ag02          1.55 eA-3
PLAT971_ALERT_2_C Check Calcd Resid. Dens.  0.86Ang From Ag02          1.53 eA-3
PLAT977_ALERT_2_C Check Negative Difference Density on H017      .      -0.42 eA-3
```

Alert level G

```
PLAT004_ALERT_5_G Polymeric Structure Found with Maximum Dimension          1 Info
PLAT083_ALERT_2_G SHELXL Second Parameter in WGHT  Unusually Large      23.94 Why ?
PLAT154_ALERT_1_G The s.u.'s on the Cell Angles are Equal ..(Note)      0.002 Degree
PLAT242_ALERT_2_G Low    'MainMol' Ueq as Compared to Neighbors of       C011 Check
PLAT242_ALERT_2_G Low    'MainMol' Ueq as Compared to Neighbors of       C012 Check
PLAT242_ALERT_2_G Low    'MainMol' Ueq as Compared to Neighbors of       C013 Check
PLAT242_ALERT_2_G Low    'MainMol' Ueq as Compared to Neighbors of       C014 Check
PLAT242_ALERT_2_G Low    'MainMol' Ueq as Compared to Neighbors of       C015 Check
PLAT242_ALERT_2_G Low    'MainMol' Ueq as Compared to Neighbors of       C01B Check
PLAT434_ALERT_2_G Short Inter HL..HL Contact F00C     ..F00P     .       2.79 Ang.
                                              1+x,1+y,1+z  =      1_666 Check
PLAT434_ALERT_2_G Short Inter HL..HL Contact F00E     ..F00S     .       2.83 Ang.
                                               -x,1-y,1-z  =      2_566 Check
PLAT720_ALERT_4_G Number of Unusual/Non-Standard Labels ..........         56 Note
             Ag01    Ag02    Ag03    F004    F005    F006    F007    F008
             F009    O00A    F00B    F00C    F00D    F00E    F00F    F00G
```

```
              N00H    F00I    N00J    F00K    N00L    N00M    F00N    N00O
              F00P    F00Q    N00R    F00S    C00T    H00T    C00U    C00V
              C00W    C00X    C00Y    H00Y    C00Z    H00Z    C010    C011
              C012    C013    C014    C015    C016    C017    H017    C018
              C019    H01A    H01B    H01C    C01A    H01D    C01B    C01C
PLAT883_ALERT_1_G Absent Datum for _atom_sites_solution_primary ..     Please Do !
PLAT912_ALERT_4_G Missing # of FCF Reflections Above STh/L=  0.600        303 Note
PLAT933_ALERT_2_G Number of HKL-OMIT Records in Embedded .res File          7 Note
                5-12  6,   4-13  5,   3-13  1,   0-14  9,   7-14  2,   0-11  4,
                6-11  6,
PLAT941_ALERT_3_G Average HKL Measurement Multiplicity ..........        2.1 Low
PLAT969_ALERT_5_G The 'Henn et al.' R-Factor-gap value ..........      3.408 Note
              Predicted wR2: Based on SigI**2  4.73 or SHELX Weight 15.16
PLAT978_ALERT_2_G Number C-C Bonds with Positive Residual Density.          0 Info
```

```
   0 ALERT level A = Most likely a serious problem - resolve or explain
   0 ALERT level B = A potentially serious problem, consider carefully
  15 ALERT level C = Check. Ensure it is not caused by an omission or oversight
  18 ALERT level G = General information/check it is not something unexpected

   3 ALERT type 1 CIF construction/syntax error, inconsistent or missing data
  21 ALERT type 2 Indicator that the structure model may be wrong or deficient
   5 ALERT type 3 Indicator that the structure quality may be low
   2 ALERT type 4 Improvement, methodology, query or suggestion
   2 ALERT type 5 Informative message, check
```

Validation response form

Please find below a validation response form (VRF) that can be filled in and pasted into your CIF.

```
# start Validation Reply Form
_vrf_PLAT042_lcz022
;
PROBLEM: Calc. and Reported MoietyFormula Strings  Differ     Please Check
RESPONSE: ...
;
_vrf_PLAT342_lcz022
;
PROBLEM: Low Bond Precision on  C-C Bonds ..............    0.01418 Ang.
RESPONSE: ...
;
_vrf_PLAT906_lcz022
;
PROBLEM: Large K Value in the Analysis of Variance ......      4.953 Check
RESPONSE: ...
;
_vrf_PLAT911_lcz022
;
PROBLEM: Missing FCF Refl Between Thmin & STh/L=    0.600         36 Report
RESPONSE: ...
;
_vrf_PLAT971_lcz022
;
PROBLEM: Check Calcd Resid. Dens.  1.02Ang From Ag01            1.93 eA-3
RESPONSE: ...
```

```
;
_vrf_PLAT977_lcz022
;
PROBLEM: Check Negative Difference Density on H017      .      -0.42 eA-3
RESPONSE: ...
;
# end Validation Reply Form
```

It is advisable to attempt to resolve as many as possible of the alerts in all categories. Often the minor alerts point to easily fixed oversights, errors and omissions in your CIF or refinement strategy, so attention to these fine details can be worthwhile. It is up to the individual to critically assess their own results and, if necessary, seek expert advice.

PLATON version of 17/09/2025; check.def file version of 17/09/2025

---

duplicate check

No duplication found

Datablock lcz022 - ellipsoid plot

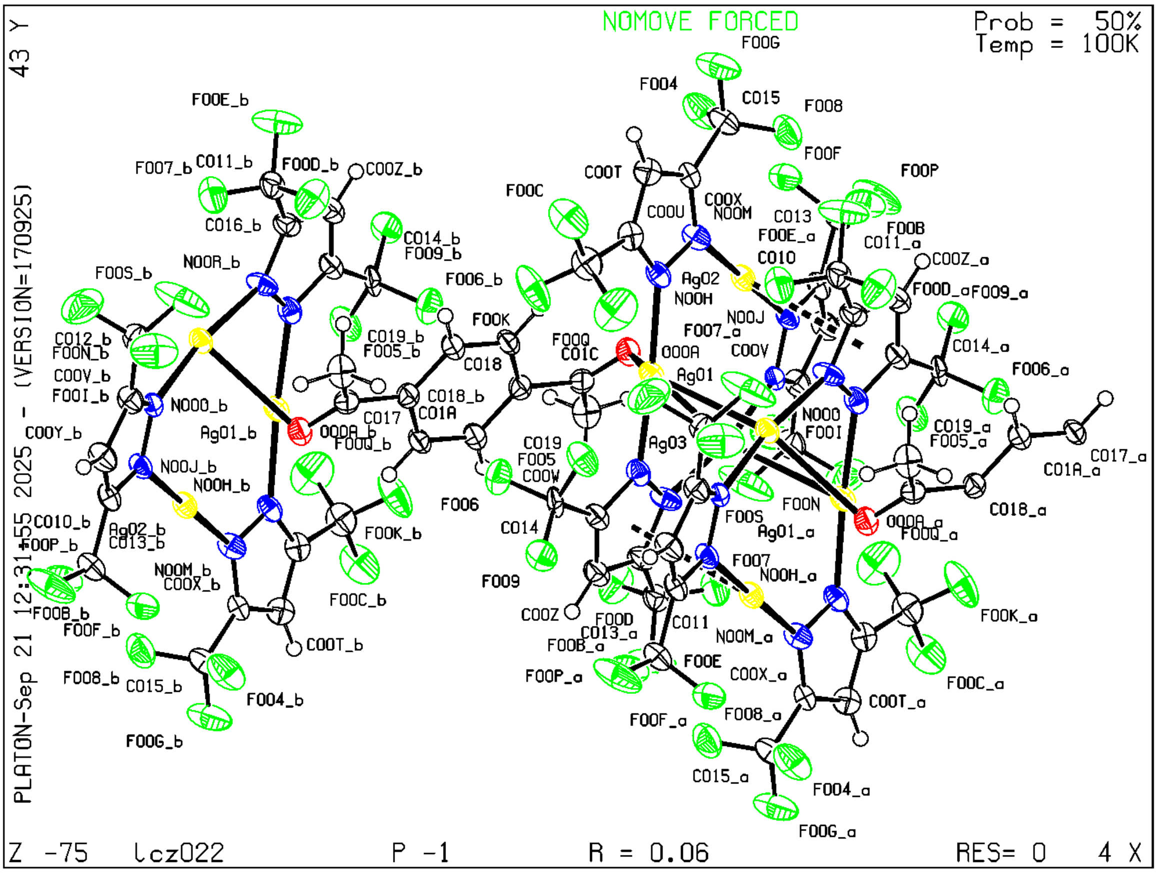

Supplement: Supplementary file 2 — Supporting File 2: anie72462‐sup‐0002‐DataFile.zip. [file ANIE-65-e1218503-s002.zip › supplementary_materials_crystallographic_data submit.zip!/cif_and_checkcif(1-114)/Ag3Pz3_22/Ag3Pz3_22_checkcif.pdf]
